# Aging epigenome begins to change in age-related neurodegenerative diseases

**DOI:** 10.4103/NRR.NRR-D-25-00805

**Published:** 2025-09-29

**Authors:** Adam Zaretsky, Debra Toiber

**Affiliations:** Department of Life Sciences, Ben-Gurion University of the Negev, Beer Sheva, Israel (Zaretsky A, Toiber D); The Brain Sciences School, Ben-Gurion University of the Negev, Beer Sheva, Israel (Zaretsky A, Toiber D)

With the rapid increase in the aging population comes a rise in the incidence and prevalence of neurodegenerative diseases. Therefore, it is critical to understand the molecular changes that occur, which can either cause disease or make brains resilient. Epigenetic changes are a common suspect and target, not only because they are among the hallmarks of aging, but also because they are flexible and could potentially be reversed.

Epigenetic modifications enable the expression of important neural genes while repressing unnecessary ones. These marks are chemical modifications, such as DNA methylation, histone post-translational modifications, or by long noncoding RNA associations and topological barriers (Houston et al., 2013). Then chromatin remodelers have the capability to alter the epigenetic state by adding or removing these chemical modifications, thereby influencing how approachable genes are to transcription machinery.

During brain development, cells acquire their unique topological DNA structure, allowing neurons to function. However, even during adulthood, certain flexibility in specific regions and modifications is allowed to facilitate learning, coping in different situations, and adjusting to the environment (Houston et al., 2013). Nevertheless, as we age, and particularly in age-related neurodegenerative diseases, the aging epigenome change, sometimes reverting to a less differentiated state and sometimes allowing the expression of non-relevant genes, resulting in cellular malfunction and a waste of energy and resources (Saul et al., 2021).

**Epigenetic regulators in neural aging:** In the context of neural gene expression, the RE-1 silencing transcription factor (REST) is an essential transcription factor that gatekeeps neural genes. When REST binds to its target gene, it recruits other chromatin remodelers that compact the chromatin, thereby preventing unwanted transcription (Lee et al., 2018). During development, REST is highly expressed in neural progenitor cells and non-neural tissues to prevent premature or ectopic neuronal differentiation (Bithell, 2011). In the mature neuron, REST plays a neuroprotective role and has been found to be highly expressed in cognitively intact elderly individuals (Lu et al., 2014). However, in neurodegenerative diseases such as Alzheimer’s disease (AD), REST is also highly expressed. Curiously, despite its heightened expression, REST fails to properly localize and execute its repressive functions in neurodegenerative patients, presenting a fascinating paradox (Meyer et al., 2019).

Another example of an epigenetic regulator is Sirtuin 6 (SIRT6), a histone deacetylase and ADP-ribosyl transferase that plays a role in a myriad of cellular functions crucial to the aging cell, such as DNA damage repair and regulation of gene expression (Onn et al., 2020). The relevance of SIRT6 to aging emerged with the development of a full-body knockout mouse model, which exhibited a progeroid (progeria-like) phenotype and early death from hypoglycemia (Mostoslavsky et al., 2006). When SIRT6 was removed specifically from the brain, mice showed impairment of learning capabilities and accumulation of phosphorylated tau, presenting AD-like pathology (Kaluski et al., 2017). In human aging brains, SIRT6 levels decline, and even more so in AD. This makes SIRT6 a critical protective factor for neural health in aging individuals (Kaluski et al., 2017).

**Figure 1:**
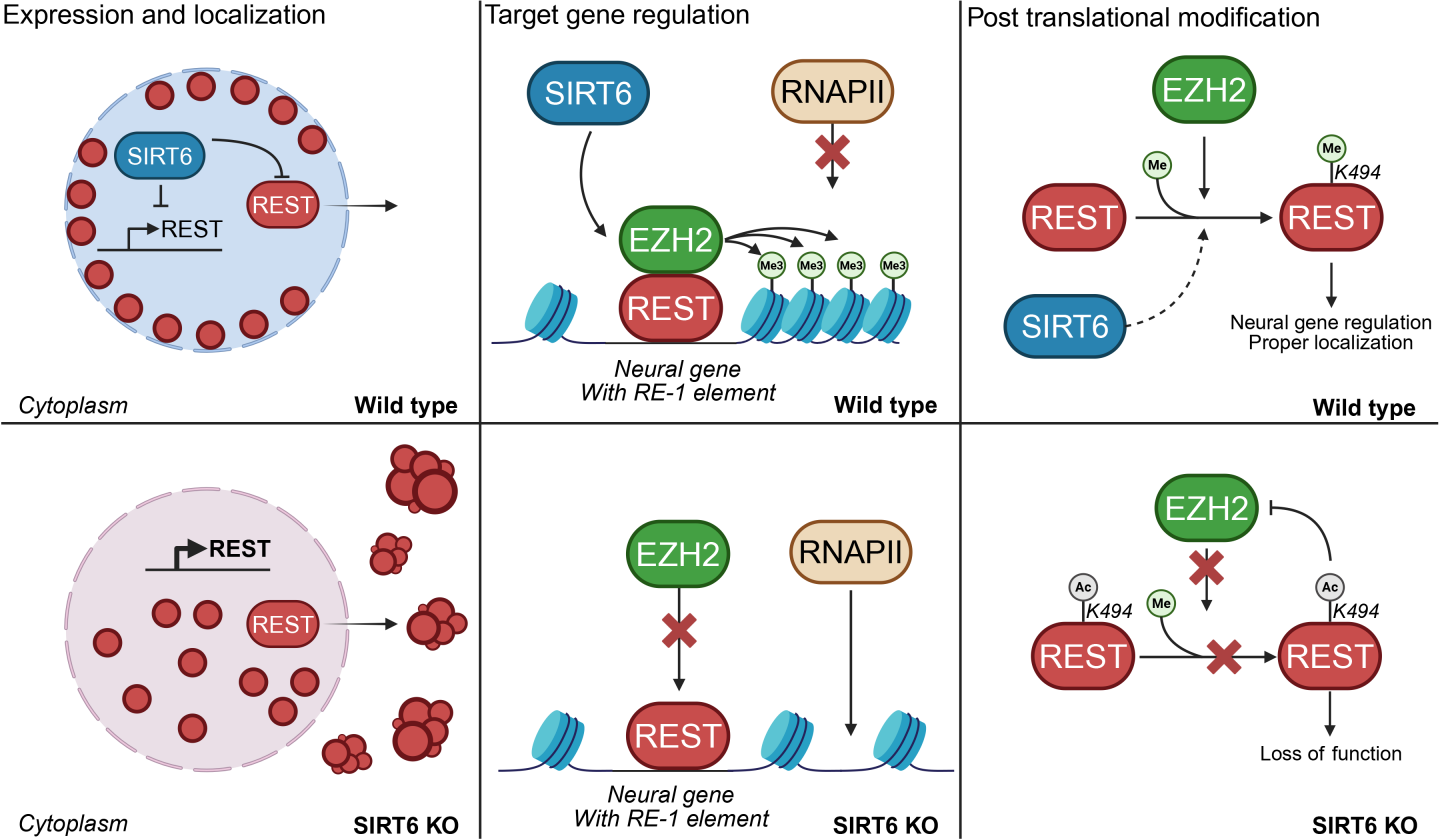
REST and SIRT6. Upper panel: Healthy chromatin with REST localized to the nuclear lamina, preventing ectopic gene expression. REST activity is regulated by SIRT6 and an EZH2-dependent methylation/acetylation switch. Lower panel: In the absence of SIRT6, REST accumulates but is mislocalized and inactive due to increased acetylation, reduced interaction with EZH2, and aggregate formation. EZH2: Enhancer of zeste homolog 2; KO: knockout; REST: RE-1 silencing transcription factor; SIRT6: sirtuin 6.

**SIRT6 and REST — a functional axis:** Brain development and healthy aging are tightly regulated by transcription factors such as SIRT6 and REST. Both proteins are crucial for proper cellular differentiation, the regulation of neural genes, and the prevention of neurodegeneration. Interestingly, while SIRT6 levels decrease with age and in neurodegenerative conditions, REST exhibits an opposing trend, with increased expression (**Figure 1**).

Recent findings suggest that SIRT6 and REST are not just independent protective entities in aging; instead, they intersect at multiple molecular levels (Zaretsky et al., 2024). SIRT6 has been shown to regulate REST expression, its subcellular localization, its interaction with chromatin-modifying enzymes, and critical post-translational modifications. Notably, under SIRT6-deficient conditions, REST becomes overexpressed yet functionally impaired. This paradoxical state is characterized by the persistent expression of REST target genes despite increased REST levels. This phenomenon reflects a loss of transcriptional repression due to disrupted chromatin interactions, particularly with the polycomb repressive complex 2 (PRC2) member enhancer of zeste homolog 2 (EZH2).

Further evidence from brain-specific SIRT6 knockout mice shows that upregulated transcripts are significantly enriched for REST targets, especially those involved in neuronal activity and differentiation. Similar patterns are observed in AD models and patient-derived induced pluripotent stem cells, where REST target genes become derepressed in parallel with increased neural excitability and accelerated neural differentiation. Although DNA binding of REST remains largely intact, its repressive function is compromised due to a reduction in EZH2 recruitment and diminished H3K27me3 deposition at REST-bound loci.

Mechanistically, this loss of function is associated with the post-translational modification of REST at lysine 494 (K494), a residue known to be methylated by EZH2. In SIRT6-deficient systems, REST is hyperacetylated at K494, a modification that interferes with methylation and disrupts REST-EZH2 interactions. This modification imbalance impairs the localization of REST to lamina-associated heterochromatin and leads to the cytoplasmic aggregation of REST in condensate-like bodies, some of which co-localize with autophagosomes. These aggregates persist even after the reintroduction of SIRT6, suggesting that the mislocalization phenotype may become irreversible.

Importantly, expression of a methylation-mimetic REST mutant (K494M) is sufficient to restore the repression of REST target genes, even in the absence of SIRT6, highlighting the functional importance of this modification. In contrast, acetylation-mimetic or non-modifiable variants fail to rescue repression and remain mislocalized. These findings position the SIRT6-REST-EZH2 axis as a central regulatory pathway in maintaining the epigenetic homeostasis of neural genes during aging and implicate the post-translational modulation of REST as a potential therapeutic target in neurodegenerative diseases.

**Therapeutic perspectives:** The broader implications of the SIRT6-REST interaction are significant for understanding neural vulnerability during aging and in disease. In normal aging, REST is upregulated as a protective response, suppressing genes that promote excitotoxicity, cell death, and protein misfolding. However, in pathological aging, such as in AD and Parkinson’s disease, REST is frequently mislocalized to the cytoplasm, often in aggregates with tau or alpha-synuclein, rendering it inactive. The loss of SIRT6 under these conditions directly contributes to this dysfunction by disrupting the acetylation-methylation switch at K494.

The balance between acetylated and methylated REST forms a binary decision point in the cell’s transcriptional logic. REST-K494Me acts as a functional repressor localized at heterochromatin boundaries, enabling the suppression of neurotoxic transcriptional programs. Conversely, REST-K494Ac loses its spatial organization, fails to recruit EZH2, and accumulates in a non-functional and potentially deleterious state. This epigenetic tipping point is regulated by the presence or absence of SIRT6. The metaphor of REST as a “brake” on neural activity is apt: SIRT6 determines whether that brake is engaged via methylation or left disengaged through unchecked acetylation.

Experimental models have demonstrated that both SIRT6 and REST play protective roles in preventing neurodegeneration. Brain-specific deletion of either gene in mice results in increased neuronal loss, gliosis, and cognitive impairment. Conversely, restoring SIRT6 activity or mimicking REST methylation can reduce amyloid and tau pathology and improve memory in AD models.

These mechanistic insights also offer tangible clinical relevance. Compounds that enhance SIRT6 activity, such as nicotinamide riboside and nicotinamide mononucleotide, or specific SIRT6 activators such as MDL-800, have shown promise in preclinical models. Likewise, targeting REST acetylation or using methylation-mimetic REST therapies may help restore repressive chromatin states and reduce neural hyperactivity. These approaches may pave the way for therapeutic strategies aimed at delaying or even reversing neurodegenerative decline.

In addition to their roles in neurodegeneration, both proteins influence neural plasticity and regeneration. SIRT6 supports DNA repair in neural progenitor cells and modulates inflammatory responses in the neural stem cell niche, while REST may help maintain the integrity of neural stem cell pools by preventing premature senescence. Their combined activity likely supports an environment conducive to neurogenesis and circuit remodeling, particularly in the hippocampus.

Cognitively, elevated REST levels have been linked to resilience in aging. In elderly individuals with high REST expression, cognition is preserved even in the presence of AD pathology, whereas a failure to upregulate REST predicts rapid cognitive decline. SIRT6 loss, by impairing REST function, may accelerate this decline by unleashing transcriptional programs that promote synaptic dysfunction, oxidative stress, and metabolic imbalance. Therefore, an increase in REST alone may not be sufficient if it is not accompanied by SIRT6 expression.

These findings suggest a revised model of transcriptional regulation in the aging brain, wherein REST abundance alone is not protective unless paired with SIRT6-mediated deacetylation. Therapeutically, boosting SIRT6 with small-molecule activators or NAD^+^ precursors, or specifically targeting acetylation state of REST, may offer new tools to delay or reverse neurodegenerative processes.

*This work was funded by the David and Inez Myers Foundation, the Israeli Ministry of Science and Technology (MOST), and The Israel Science Foundation (No. 422/23) (to DT).*
